# One Is the Coldest Number: How Group Size and Body Weight Affect Thermal Preference in Weaned Pigs (3 to 15 kg)

**DOI:** 10.3390/ani11051447

**Published:** 2021-05-18

**Authors:** Lindsey A. Robbins, Angela R. Green-Miller, Jay S. Johnson, Brianna N. Gaskill

**Affiliations:** 1 Department of Animal Sciences, Purdue University, West Lafayette, IN 47906, USA; bgaskill@purdue.edu; 2 Department of Agricultural and Biological Engineering, University of Illinois at Urbana-Champaign, Urbana, IN 61801, USA; angelag@illinois.edu; 3 USDA-ARS, Livestock Behavior Research Unit, West Lafayette, IN 47906, USA; Jay.Johnson@ars.usda.gov

**Keywords:** thermal preference, pigs, thermal comfort zone, social aggregation

## Abstract

**Simple Summary:**

Exposure to thermal stress can negatively impact an animals’ overall welfare, resulting in decreased body condition, slower growth rates, and in severe cases, mortality. Understanding the thermal comfort of pigs can help producers reduce thermal stress and improve the overall well-being of these animals. To understand pigs’ thermal comfort, this study utilized temperature preference with weaned pigs by allowing them to select from a range of temperatures within a thermal apparatus. However, temperature preference is complicated given that a variety of factors can influence thermal comfort. Previous research has indicated that temperature preference is altered based on the number of individuals tested as this can alter their thermal comfort. Social aggregation, through huddling, results in greater heat conservation and animals find cooler temperatures more comfortable. Thus, this study looked at how social groups and different body weight could influence the temperature preference of pigs. Results showed that individual pigs preferred warmer temperatures compared to those in groups of 2 and 4, and that heavier pigs preferred cooler temperatures compared to medium- and lightweight pigs. This study demonstrates that a greater number of individuals perceive a cooler temperature as being within their comfort zone, whereas an individual does not have access to the thermal benefits of social aggregation.

**Abstract:**

Housing pigs within their thermal comfort zone positively impacts productivity and performance. However, fundamental information on behavioral thermoregulatory responses of individual and group-housed pigs is meager. As a gregarious species, pigs prefer to be near one another, touching and often huddling. As pigs huddle together, they decrease their heat loss to the environment by decreasing exposed surface area and increasing mass. Additionally, pigs gain weight rapidly as they age. As an individual grows, their ability to withstand lower temperatures increases. We hypothesized that group size would alter pig thermal preference and that thermal preference would change based upon body weight. Thirty-six groups of pigs (n = 2 pigs/group) were tested in a factorial design based on group size (1, 2, or 4) and weight category (small: 5.20 ± 1.15 kg; medium: 8.79 ± 1.30 kg; and large: 13.95 ± 1.26 kg) in both sexes. Treatment groups were placed inside a chamber with a controlled thermal gradient (4.6 m × 0.9 m × 0.9 m; L × W × H) that ranged in temperature from 18 to 30 °C. Pigs habituated to the gradient for 24 h. The following 24 h testing period was continuously video recorded and each pig’s location during inactivity (~70% daily budget) within the thermal apparatus was recorded every 10 min via instantaneous scan sampling. Data were analyzed using a GLM and log10 + 0.001 transformed for normality. Tukey tests and Bonferroni-corrected custom tests were used for post hoc comparisons. Peak temperature preference was determined by the maximum amount of time spent at a specific temperature. Both group size (*p* = 0.001) and weight category (*p* < 0.001) influenced the thermal location choice of pigs. Individual pigs preferred 30.31 °C, which differed from a group of 2 (20.0 °C: *p* = 0.003) and 4 pigs (20.0 °C: *p* < 0.001). The peak temperature preference of the small pigs (30.2 °C) differed from the large pigs (20.0 °C: *p* < 0.001) but did not differ from the medium-sized pigs (28.4 °C: *p* > 0.05). Overall, heavier pigs and larger groups preferred cooler temperatures.

## 1. Introduction

Pigs achieve thermal balance through a combination of physiological and behavioral processes including panting, huddling and thermotaxis [1,2]. Pigs will seek out their preferred ambient temperature, where they will not have to utilize any physiological mechanisms for thermoregulation and there is minimal heat exchange between the animal and its environment [2]. Heat loss is affected by the thermal gradient between the animal, the ambient temperature, and its thermal conductance [3]. Overall, thermal conductance is dependent on both the total surface area of the animal exposed to its environment and its insulation properties [4]. The surface area to mass ratio will vary among animals depending on their body weight. However, as the body mass increases, the surface area does not increase proportionately [5]. Thus, the surface to mass ratio is higher for smaller pigs compared to heavier pigs [6].

Not only does body weight affect the surface area to mass ratio, but behavior, such as huddling, can alter the other side of the ratio. When exposed to cold stress (CS), pigs will huddle in a close-packed group, reducing the amount of an individual’s surface area exposed to the environment. Reducing the surface area exposure consequently decreases the amount of heat loss [5]. Unfortunately, the benefits of huddling do not linearly increase with more individuals. There is a critical number at which the addition of more individuals to the huddle will provide negligible benefits [7].

The surface area to mass ratio of an individual, as described above, directly affects heat loss and conservation. These factors will affect an animal’s thermal preference [2]. An animal is expected to seek ambient temperature in their comfort zone and will move if the condition becomes too cool or too warm. The animal is expected to exhibit selection behavior, when possible, to manage its thermal environment by choosing a temperature where it does not need to use any major physiological mechanisms to heat or cool itself. 

The thermal preferences of piglets have been previously studied; however, these studies have focused on the choices of either individual piglets [8,9] or extremely young (20 to 72 h old) and lightweight piglets [10,11]. While this information is important for understanding basic thermoregulatory processes and choice, there remains a need to better understand the thermal needs of post-weaned pigs, which are extremely gregarious and are typically housed in groups in a production setting. An understanding of how group size (surface area exposure) and body weight (mass) affects the temperature preference of young pigs would aid in the development of temperature recommendations for both producers and researchers. 

The purpose of the current study was to determine the thermal preference of young pigs in various group sizes and weight categories. We hypothesized that group size would alter thermal preference and that this would vary by body weight. Specifically, surface area to mass ratio would be reduced in bigger groups and with heavier pigs, thus reducing overall heat loss and resulting in cooler temperature preferences.

## 2. Materials and Methods

All procedures involving animal use were approved by the Institutional Animal Care and Use Committee at Purdue University (protocol # 1901001844), and animal care and use standards were based upon the Guide for the Care and Use of Agricultural Animals in Research and Teaching (referred to as the *Ag Guide* from here on) [12]. Temperature preference testing began on 15 March 2019 and ran until 23 April 2019. Testing was conducted on the same farm but in a different building from the nursery room, where the pigs were housed. The thermal apparatuses, for preference testing, were located in the Purdue environmental room.

### 2.1. Animals and Housing

Pigs [Duroc × (Landrace × Yorkshire)] were weaned at 20.0 ± 1.3 d of age and housed in nursery room 1 at the Purdue University Animal Science Research Education Center (West Lafayette, IN, USA) until testing. A nursery pen (0.95 m × 1.43 m) held up to 8 pigs; pigs were housed single sex and within their randomly assigned treatment group. This was done to improve the ease of identifying and moving predefined testing groups from the nursery room to the testing room. The nursery room received natural lighting via windows (15:9 light:dark) and the temperature was held at 25 ± 5.8 °C with 58.1 ± 10.5% relative humidity. Pigs were given access to food and water ad libitum. Body weight was documented before weaning and pigs were randomly assigned into one of three group size categories (1, 2 or 4 pigs per group) and within similar body weight conditions. 

### 2.2. Experimental Design

To determine the number of animals needed for this study, Mead’s resource equation was used a priori for a 3-weight category × 3 group size × 2 sex × 2 replicate factorial design [13] for a total of 36 groups (Table 1). Eighty-four nursery pigs [Duroc × (York × Landrace)] were randomly selected to be tested as either an individual, in a group of 2, or 4 pigs. In addition to this, pigs were blocked by three different weight categories: small (5.20 ± 1.15 kg); medium (8.79 ± 1.30 kg); or large (13.95 ± 1.26 kg). This was achieved by placing pigs at weaning into groups of a similar weight. The pig weight groups were monitored over the course of the study and verified prior to testing. 

Pigs were transported in a cart approximately 91 m from the nursery room to the environmental room. For each temperature preference testing session, only 2 groups could be run at a given time (one in each apparatus) within the environmental room. Group size and weight category were balanced across testing runs and apparatuses. The pigs had 24 h to acclimate to the new environment and were able to freely explore the entirety of the thermal apparatus. This was followed by a 24 h testing period, which was used for all subsequential data analyses. Each apparatus was cleaned between acclimation and testing within groups, and subsequent testing between different groups to avoid any bias of temperature preference between acclimation and testing. During the cleaning, pigs were removed from the thermal apparatus and placed in stalls within the same environmental room for approximately 2 h to clean and re-establish the thermal gradient. Waste was removed with a pressure washer and the apparatus floors and walls were disinfected (LYSOL disinfectant all-purpose cleaner, Reckitt Benckiser LLC, Parsippany, NJ, USA). After cleaning, pigs were returned to their assigned thermal apparatus after the thermal gradient was stable. The gradient was considered stable when 3 readings, measured every 15 min, did not vary by more than 0.2 °C [14]. During experimental set-up and testing, researchers were not blinded to the pigs’ treatments; however, video coders were blinded to weight category and sex when analyzing video data.

#### 2.2.1. Thermal Apparatus

**Figure 1:**
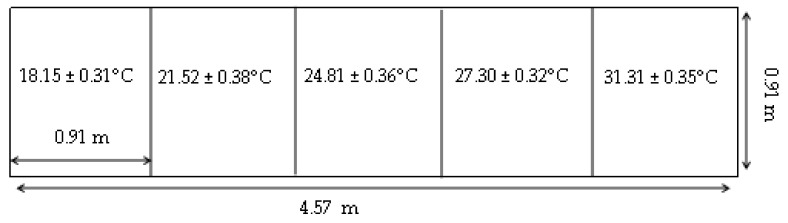
Scale drawing of one of two thermal apparatuses with thermal zones (mean ± SD temperature within each zone). Data loggers were placed 0.40 m and were in the middle of each thermal zone to calculate the average temperature of each zone.

**Figure 2:**
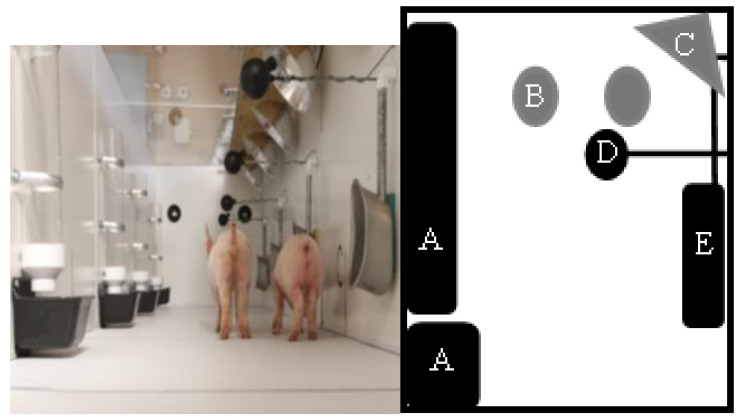
Image on the left displays of one thermal apparatus with a group of 2 piglets. On the right, a diagram depicts what was inside the thermal apparatus: (A) feeders with PVC pipe to allow gravity feeding found within each thermal zone (0.40 m apart), (B) two computer fans that ducted cold air into the thermal apparatus located 0.60 m from the ground, (C) heating elements inside a heat guard, (D) 3” black globe with data logger probe measuring temperature every 15 min located in the middle of each thermal zone 0.40 m apart, and (E) waterers with ad libitum access located 0.40 m apart and within each thermal zone. A total of 5 feeders, waterers and black globes, one per thermal zone, were within each thermocline.

Based on previous work with thermal preference [14,15], two identical thermal apparatuses were constructed. The thermal apparatus temperature zones were designed based on the *Ag Guide* recommendations for thermal conditions of pigs weighing between 3 and 15 kg [12]. The thermal apparatus contained a gradient between 18.2 ± 0.3 and 30.3 ± 0.5 °C (Figure 1). The thermal apparatus was designed to allow pigs to freely walk between and choose between a range of temperatures which encompassed temperatures around the recommendations in the *Ag Guide* (26 to 32 °C) to determine whether a shift in thermal preference can be observed [14,15]. The thermal apparatus measured 4.57 m × 0.91 m × 0.91 m (L × W × H; Figure 1). Pigs had access to water from waterers (AquaChief™, Hog Slat, Inc. New Grove, NC, USA) and food provided in goat feeders (14.2 L: 15.88 cm × 8.20 cm × 7.32 cm) ad libitum. When goat feeders emptied, they were re-supplied by gravity from a clear PVC pipe above the feeder (5.08 cm diameter, 30.48 cm length). These feeders and waterers were placed every 0.76 m, such that each thermal zone contained one feeder and one waterer (Figure 2). Each apparatus had a Plexiglas lid (MFG-Acrylic 1.52 m × 1.91 cm × 0.61 m: Meyer Plastics, Inc., Lafayette, IN, USA) to allow the pigs to be video recorded. The environmental room lights were set on a timer with a 12:12 light:dark cycle (light on at 0800 and off at 2000 h); however, this room received natural lighting via windows (15:9 light:dark). 

The temperature gradient was created by heating elements (100 W and 150 W reptile ceramic infrared brooder bulbs, Aiicioo, Shenzhen, China) that added incremental amounts of heat as the air moved down the gradient. Ventilation air entered the chamber via a conditioning box from the cool end of the chamber and exited through exhaust fans at the warm end of the chamber. The conditioning box (0.61 m × 0.61 m × 0.46 m, L × W × H; Figure S1) was located above the two thermal apparatuses and cooled the supply air before directing into the cool end of the chamber.

A portable air conditioning unit (24,500 BTU, LG Electronics, Seoul, South Korea) was wired to a CoolBot (Store It Cold LLC, North Fort Myers, FL, USA) which controlled the air conditioning unit to cool the supply air to 15 ± 0.81 °C. Two eight-inch duct fans (99.1 L/s airflow, Suncourt Inc., Durant, IA, USA) per thermal apparatus were used to pull the air from the conditioning box into the thermal apparatuses to create the temperature gradient. To avoid any aversion of the pigs to airflow speed, both chambers maintained a low-velocity airflow (Alnor air velocity meter, AVM410 FLW Inc., Huntington Beach, CA, USA; range of 0 to 20 m/s with accuracy of ±5% of reading or ±0.025 m/s with 0.01 m/s resolution). The temperature was monitored using data loggers (HOBO Data Logger; U12-013, Onset Computer Corporation, Bourne, MA, USA; temperature range of −20 to 70 °C with accuracy of ±0.35 °C and relative humidity range 5–95% with accuracy of ±2.5% to max 3.5%) with temperature probes to assure that the two apparatuses were as identical as possible.

#### 2.2.2. Behavior and Posture by Location

Pigs were continuously video recorded during the 24 h testing period for behavior, location, and posture data collection. Infrared cameras (Sony Corporation, Tokyo, Japan) provided both day and night video, which was recoded with video surveillance software (GeoVision, Taipei City, Taiwan). The scan sample interval was determined by comparing different sampling intervals (continuous, 5, 10, 15, 20, 25, 30 min) based on the frequency of time spent at each location. Data from each subsample were compared using information from both the intercept and slope provided from a regression analysis pairwise comparison and considered to accurately estimate the behavior or posture if the intervals were not significantly different from each other compared to the continuous sampling [16]. Pig location in a thermal zone, behavior (Table S1), and posture (Table S2) were recorded using instantaneous scan samples every 10 min [14]. If pigs were observed in more than one thermal zone (location), the percentage of the pig in each zone was documented in 25% increments (head, front quarter, mid-section and rump; Figure S2) [14].

The proportion of behaviors was calculated for each group of pigs by counting the total number of times each behavior category was observed in each location per day. This was then totaled per group and divided by the total number of observations. This calculation was repeated for posture. Although other behaviors were coded, only inactive behavior was analyzed since pigs spent approximately 70% of their time performing this behavior.

### 2.3. Analyses

All analyses were performed using the PROC MIXED (GLM) procedure in SAS 9.4 (SAS Institute INC., Cary, NC, USA). The assumptions of the GLM (normality of error, homogeneity of variance, and linearity) were confirmed post hoc, and data were transformed when necessary to meet these assumptions [17]. The threshold for significance *p* < 0.05 was used and Bonferroni corrected where applicable.

A cubic regression model was performed on the proportion of time for both posture and behavior data. This was then transformed where necessary to meet the assumptions of a GLM. To avoid pseudoreplication and accommodate repeated measures, analyses were blocked by the group of pigs nested within sex, group size and weight category. Pig group was treated as a fixed effect, as there is no meaningful wider population of pigs representing the group size and weight category combinations, to which the results could pertain [18].

#### 2.3.1. Behavior by Location

The behavior analysis only included the proportion of time pigs were inactive. Main effects and second-order interactions of sex, group size, weight category, and location were tested with a cubic variable of location. The three-way interaction between group size, weight categories and temperature were also included in this model. The cubic curve (see Figure S3 for equations) from the final model above was generated in 0.2 °C increments starting with the coldest thermal zone temperature (18.2 °C) and increasing to the warmest temperature (30.4 °C). The peak temperature preference was identified by determining the location (temperature) where pigs spent most of their time based on the regression model. The temperature preference range was then calculated from the peak temperature ± SE. In each thermal zone, Tukey tests were used to determine LSM differences within group size and weight category. Since Tukey tests were run 5 times (for each thermal zone), the alpha was Bonferroni corrected for the multiple tests (α = 0.05/5 = 0.01).

#### 2.3.2. Posture by Location

Posture data were split into two separate models. The first model excluded individually tested pigs and evaluated huddling behavior only. This was done since individual pigs are incapable of performing huddling. This analysis was originally run as a cubic regression model but was reduced to a linear model, since quadratic and cubic terms were not significant. Main effects and second-order interactions of sex, group size, weight category, and location were tested. In addition, the three-way interaction of group size, weight category, and temperature was also evaluated. Data were square root transformed to meet the assumptions of a GLM. The second posture model included all group size treatments and analyzed all postures except for huddling. A cubic regression model was used with data log10 + 0.001 transformed to meet the assumptions of a GLM.

## 3. Results

To improve readability, test statistics from our main models will be provided in Supplementary Tables and just *p*-values will be presented in the paragraphs below. However, test statistics for post hoc tests will provided. 

### 3.1. Behavior by Location

**Figure 3:**
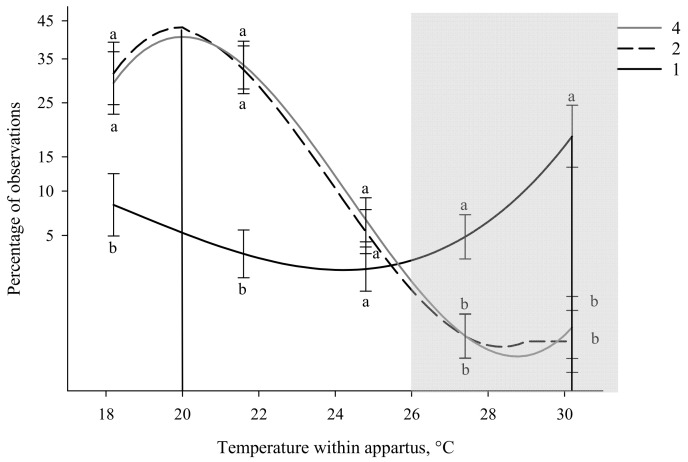
Percentage of observations in different temperatures within the thermal gradient while inactive based on group size. The effects of being tested as an individual (1), in a group of 2 or 4, on temperature preference. Standard error bars are located at the temperatures of the five thermal zones (18.2, 21.5, 24.8, 27.3, and 30.3 °C) and different letters denote significant Tukey pairwise comparisons (*p* < 0.01). The gray box indicates the recommended temperatures (26 to 32 °C) for pigs between 3 and 15 kg [12]. Equations for the curves shown above can be found in Figure S3A.

During times of inactivity, group size (*p* = 0.001) influenced where pigs spent their time (Table S3). Individual pigs preferred a warmer peak temperature (30.2 °C) compared to pigs tested in a group of 2 (F_1,127_ = 10.95; *p* = 0.001; 20.2 °C) and 4 (F_1,127_ = 9.73; *p* = 0.002; 20.0 °C, Table 2; Table S3). No differences in peak temperature preference was observed between pigs tested in a group of 2 compared to a group of 4 (F_1,127_ = 0.04; *p* = 0.852, Figure 3).

Pigs tested in a group of 2 had a temperature preference range of 18.8–21.0 °C and groups of 4 preferred 18.8–21.2 °C (Table 2). No preferred temperature range could be calculated for pigs tested individually because individual piglets do not demonstrate a peak temperature nor a preferred temperature range (Figure 3). Individual pigs spent less time in cooler thermal zones (18.2 and 21.5 °C) compared to pigs tested in a group of 2 (*p* = 0.002 and <0.001, respectively) and 4 (*p* = 0.002 and 0.004, respectively; Figure 3). Individual pigs also spent more time at 30.31 °C compared to those tested as a group of 2 and 4 (*ps* ≤ 0.001; Figure 3). No difference between group size was observed at 24.8 °C, nor was there any difference observed between pigs tested as a group of 2 and 4 at any thermal zone (Figure 3). 

**Figure 4:**
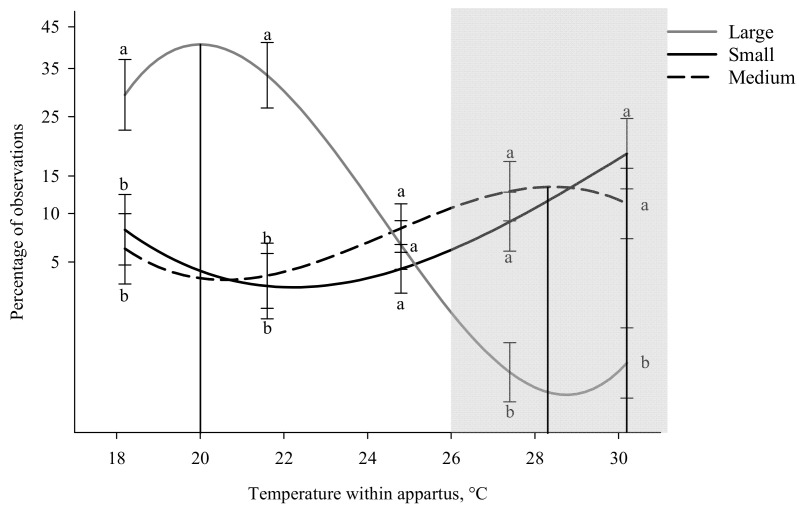
Percentage of observations in different temperatures within the thermal gradient while inactive based on weight category. The effects of being tested as a small group (3–7 kg), medium (7.1–11 kg) and large (11.1–15 kg), on temperature preference. Temperature within the thermal apparatus is plotted on the *x* axis and percentage of time spent are plotted on the *y* axis as a square root scale. Cubic peaks are indicated by vertical lines corresponding to group weight category. Standard error bars are located at the temperatures of the five thermal zones (18.2, 21.5, 24.8, 27.3, and 30.3 °C) and different letters denote significant Tukey pairwise comparisons (*p* < 0.01). The gray box indicates the recommended temperatures (26 to 32 °C) for pigs between 3 and 15 kg [12]. Equations for the curves shown above can be found in Figure S3B.

In addition to group size, during times of inactivity weight category (*p* < 0.001) influenced where pigs spent their time (Table S3). Small-weight-category pigs preferred a warmer temperature (30.2 °C) than those in the large-weight category (20.0 °C: F_1,127_ = 20.62; *p* < 0.001) but did not differ from the medium category pigs (28.4 °C; Table 2). Large-weight-category pigs’ peak thermal preference did differ from the medium category pigs (F_1,127_ = 24.26; *p* < 0.001; Table 2; Table S3). Large-weight-category pigs spent more time in the 18.2 and 21.5 °C zones compared to both small- (*p* = 0.015 and <0.001, respectively) and medium-weight-category pigs (*p* = 0.008 and <0.001, respectively; Figure 4). Medium- and small-weight-category pigs spent more time in the 27.3 and 30.3 °C zones compared to pigs in the large-weight category (*p* ≤ 0.001 for all comparisons; Figure 4). No difference between any weight category at 24.8 °C or between small- and medium-weight-category pigs at any thermal zone (Figure 4).

### 3.2. Posture by Location

A linear relationship was found between temperature and the percent of observations where pigs were observed huddling; where huddling decreased as temperatures increased (*p* = 0.009; Table S4). 

**Figure 5:**
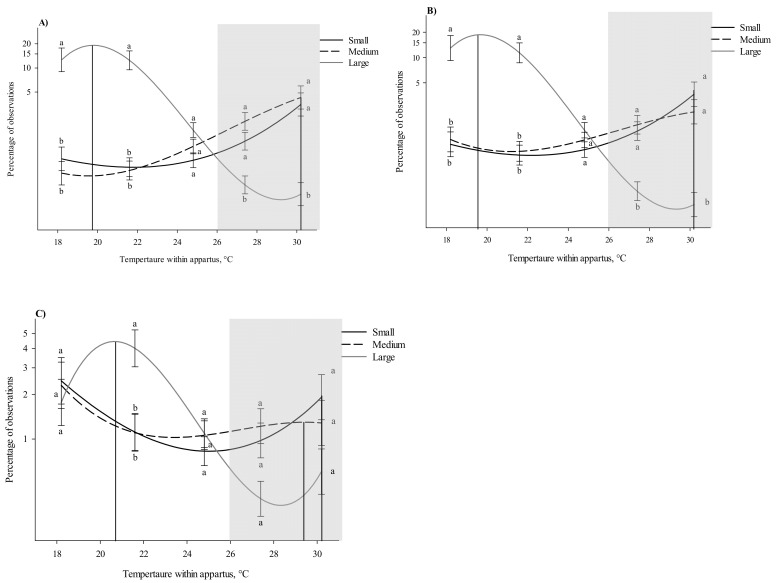
Percentage of observations in different temperatures within the thermal gradient while in different postures: (**A**) lateral laying (**B**) sternal laying and (**C**) upright. The effects of being tested by weight category: small (3–7 kg), medium (7.1–11 kg) and large (11.1–15 kg), on temperature preference based on posture. Temperature within the thermal apparatus is plotted on the *x* axis and percentage of time spent are plotted on the *y* axis as a log10 + 0.001 scale. Cubic peaks are indicated by vertical lines corresponding to group weight category within posture. Standard error bars are located at the temperatures of the five thermal zones (18.2, 21.5, 24.8, 27.3, and 30.3 °C) and different letters denote significant Tukey pairwise comparisons (*p* < 0.01). The gray box indicates the recommended temperatures (26 to 32 °C) for pigs between 3 and 15 kg [12]. Equations for the curves shown above can be found in Figure S3C.

When looking at all postures, except huddling, there was a three-way interaction observed between weight category, posture and the time spent in thermal zones (*p* < 0.001; Table S5). While in the lateral posture, small and medium pigs were observed most at a warmer temperature (30.2 and 29.4 °C, respectively) compared to large pigs (20.6° C; F_1,459_ = 6.70 and 14.80; *ps* ≤ 0.001, respectively, Table S5). In comparison to small and medium pigs, large pigs spent more time lying laterally in the 18.2 and 21.5 °C thermal zones (*ps* < 0.001; Figure 5A). Large pigs also spent less time at the warmest thermal zones (27.3 and 30.3 °C) compared to both medium (*p* < 0.001) and small pigs (*p* = 0.031 and *p* < 0.001, respectively) while in lateral position (Figure 5A). No differences between small and medium pigs were observed at any thermal zone and no difference between any weight category while in lateral posture was observed at 24.8 °C (Figure 5A).

While in the sternal posture, small and medium pigs were observed at the same peak temperature preference (30.2 °C) compared to large pigs (19.6 °C; F_1,459_ = 8.93 and 4.37; *p* = 0.037 and 0.003, respectively, Table 2). No differences in peak thermal preference was observed between medium- and small-weight-category pigs while in the sternal position (Figure 5B). While in the sternal posture, large pigs spent more time at the cooler thermal zones (18.2 and 21.5 °C) compared to both small and medium pigs (*p* < 0.001; Figure 5B). Large pigs also spent less time at the warmest thermal zones (27.3 and 30.3 °C) compared to both medium and small pigs (*p* < 0.001) in the sternal position (Figure 5B). No differences between small and medium pigs were observed at any thermal zone and no difference between any weight category while in lateral posture was observed at 24.8 °C (Figure 5A). 

Finally, while in the upright posture, small- and medium-weight-category pigs were observed at the same peak temperature of 30.2 °C, both were different compared to large pigs’ peak preference of 19.8 °C (F_1,459_ = 35.25 and 31.10; *p* < 0.001, respectively, Table 2). While in the upright posture large pigs spent the most time at 21.5 °C compared to both small (*p* = 0.038) and medium pigs (*p* = 0.033; Figure 5C). No differences were observed between small and medium pigs at any thermal zone while in the upright posture and no differences were observed at 18.2, 24.8, 27.3, and 30.3 °C between any weight category (Figure 5C). Additionally, when looking at posture, there was an interaction between group size, weight category, and time spent in various temperatures, regardless of posture (*p* < 0.001; Table S5)

## 4. Discussion

As predicted, pigs tested individually preferred warmer ambient temperatures than pigs tested in a group of 2 or 4. The peak temperature preference of individually tested pigs was 10.0 °C warmer than groups. A group of pigs have the added benefit of huddling which reduces surface area exposure to the ambient temperature. Several studies in rodents indicate the energetic benefits of huddling as a function of group size [19,20]. Huddling reduces mean energy expenditure by 31% for rodents housed in groups of 2 and 5 when exposed to 5 °C when compared to an individual. Like mice, our data support this basic phenomenon where group-housed pigs (2 or 4/group) preferred cooler temperatures than a solitary animal. 

While clear results appear between an individual animal and those housed in a group, the results indicate that there is no statistical difference in preference between a group of 2 or 4 pigs. This could be due for a variety of reasons. The first could be due to the law of diminishing returns, which states that the greatest response occurs with the initial increments of insulation, or in this case number of pigs, and the rate of return declines as more pigs are added to the group [21]. Even though this study did not test groups as large as what is seen in commercial settings, other studies may provide insight into how our data may relate to larger groups of pigs. A study done on a large groups of nursery pigs found that they preferred cooler temperatures at night compared to the day [22]. The large mass from such a big group of pigs huddling may provide enough insulation that too much heat is being retained, causing pigs to prefer cooler temperatures. Unfortunately in this study, the authors did not record huddling behavior to determine whether this may be the reason for the results they observed. Another reason we did not see differences between groups of 2 or 4 pigs could be due to the thermal zones not being refined enough. We tested a wide range of temperatures, therefore the design of this study may not have been able to identify subtle differences between these group sizes, if they exist. 

In addition to group size altering thermal preference, weight category influenced where pigs spent their time. As predicted, smaller pigs preferred warmer temperatures compared to large-weight-category pigs. The peak temperature preference of smaller pigs was 1.8 and 10.2 °C warmer than medium and large pigs, respectively. Lighter-weight animals have a larger surface area to mass ratio, which enables them to lose heat more efficiently but they also have higher energy requirements per unit of body mass compared to heavier individuals [23]. Thus, a smaller animal would prefer a warmer temperature, to minimize heat loss, compared to a larger individual [23]; which is what was observed in this study. These data indicate that as pigs age and gain weight, the nursery room temperature could be reduced periodically to stay within the growing pig’s thermal comfort zone. Previous research has suggested lowering ambient temperature for nursery pigs as they age with a 0.5 to 0.6 °C decrease per week [24]. Even though smaller pigs displayed a clear preference for warmer temperatures, this is likely negated by being housed in groups (there was no significant interaction between our treatment variables), which is common practice on farm. Perhaps a better set of data that could inform nursery room recommendations would be to evaluate the thermal preference of common group sizes used on farm and evaluate their preferences periodically while within this age range. These data highlight the importance of temperature preferences being conducted in groups rather than individuals, as previous research has primarily done. 

Though initially included in our statistical model, there was no interaction between weight category, group size, and temperature as we had predicted when looking at pigs during times of inactivity. Anecdotally, individually tested pigs in the small-weight category appeared to display escape behaviors (i.e., jumping at walls) during the acclimation phase, whereas large-weight pigs did not demonstrate this behavior as much. This could be indicative of social stress, with smaller pigs relying on social context more compared to larger-weight-category pigs. This highlights the importance of testing thermal preference in both individual and group settings, as this may alter behavior. Previous research on thermal preference in pigs has focused on the regulatory capacities of an individual [10], which is likely an oversight for such a gregarious species that are usually housed in a group setting. 

In addition to looking at behavioral thermoregulation, this study examined how thermal choice altered posture. Pigs typically take a sternal posture when in cooler temperatures to better conserve heat and a lateral posture is often observed when animals are within their thermal comfort zone [25]. However, our posture data were only significant when included in a three-way interaction with weight category and thermal preference. Across all the postures documented, small- and medium-weight-category piglets chose the warmest temperature possible in the thermal gradient. Due to this choice, we do not know if these groups would have chosen a warmer temperature if it were an option. Large pigs showed an expected preference curve, with more time spent in the cool temperatures and very little in the warmer areas. However, this curve was similar across all postures. This indicates that pigs in this large-weight category spent all their time, regardless of posture, in a particular area. 

The present study expands our knowledge of pig thermal preference and how this is altered by weight and group size. It should be noted that the gradient did not allow for observing the thermal preference of individual and small-weight-category pigs. To account for this the apparatus would need to be elongated but due to facility constraints this was not possible. Further, food was not measured due to pigs knocking food out of the bowls and in some occasions, though rare, removing the feeder from the wall. It would be of interest for future studies to look at average daily gain and food intake. Though not part of the original purpose of this study, it is worth noting that the temperature preference range of pigs in this study are lower than the recommendations found in the *Ag Guide* (26 to 32 °C) [12]. The thermal recommendations in the *Ag Guide* were based on research conducted over 30 years ago [26,27,28,29]. Since the development of these recommendations, genetic selection has focused on lean growth, which creates an animal with a higher metabolic heat production. This increased heat production could be the reason for the lower preferred temperatures found in this study, especially for heavier weights. As selection for specific traits is likely to change in the future, thermal preference should be evaluated regularly to help avoid thermal stress and improve pig welfare.

## 5. Conclusions

Overall, this study demonstrates that being housed in a group (either 2 or 4 pigs) and with a heavier body weight resulted in a cooler thermal preference in young pigs (8 to 15 kg). Pigs in groups of 2 and 4 had preferred temperature ranges of 18.6–21.0 and 18.6–21.2 °C, respectively. However, no range could be observed with individual pigs as the upper critical range, that is the warmer temperature they would have avoided, did not exist within the thermal range tested. Additionally, pigs with a medium body weight (8.79 ± 1.30 kg) preferred temperature ranges of 26.2–29.6 °C and those with heavier body weights (13.95 ± 1.26 kg) preferred a range of 19.0–21.0 °C. Like individually tested pigs, there was no range observed with small-weight pigs (5.20 ± 1.15 kg) for the same reason. Further, no interaction between group size and weight category appeared to affect thermal preference during times of inactivity. These data provide guidance on the thermal needs of 8 to 15 kg pigs when housed various group sizes. Fortunately, body weight does not seem to play a role in thermal choice when pigs are inactive (which accounts for approximately 70% of their day) and housed in groups, thus attempting to create multiple microclimates for smaller pigs is not necessary if pigs are housed in groups. 

## Figures and Tables

**Table 1:** Number of pigs and average weight exposed by weight category and group size.

Weight Category, kg (*n* = 12)	Group Size (*n* = 4)	Average Weight, kg (Average ± SD)
Small (3–7)	1	5.08 ± 1.22
	2	5.15 ± 0.73
	4	5.65 ± 1.34
Medium (7.1–11)	1	9.23 ± 1.78
	2	9.23 ± 1.11
	4	9.09 ± 2.36
Large (11.1–15)	1	12.79 ± 1.32
	2	14.67 ± 0.49
	4	14.29 ± 1.15

**Table 2:** Peak temperature preference, °C, by group size and weight category for inactive behaviors and posture (LSM ± SE) based on regression formula.

	Parameter	Peak Temperature Preference, °C	Temperature Preference Range, °C
Behavior Data	Group Size		
	1	30.2 *	N/A
	2	20.2 ^+^	18.6–21.0
	4	20.2 ^+^	18.6–21.2
	Weight Category		
	Small	30.2 *	N/A
	Medium	28.4 *	26.2–29.6
	Large	20.0 ^+^	19.0–21.0
Posture Data	Upright		
	Small		
	Medium	30.2 ^¶^	N/A
	Large	30.2 ^¶^	N/A
	Sternal	19.8 ^§^	19.0–20.6
	Small		
	Medium	30.2 ^¶^	N/A
	Large	30.2 ^¶^	N/A
	Lateral	19.6 ^§^	18.8–20.6
	Small		
	Medium	30.2 ^¶^	N/A
	Large	29.4 ^¶^	28.4–30.2
	Huddling	20.6 ^§^	19.2–21.8
	Huddling	18.2	18.2–18.8

Different symbols denote a significant difference (*p* < 0.05). N/A refers to being unable to calculate the temperature preference range due to the lower critical limit being unobserved.

## Supplementary Materials

The following are available online at https://www.mdpi.com/article/10.3390/ani11051447/s1, Table S1: Ethogram for behaviors, Table S2: Ethogram for postures, Tables S3 to S5: Statistical output, Figure S1: Image of conditioning box, Figure S2: Image of pig as sections used when coding video, and Figure S3A–C demonstrate the equation use to create the regression models seen in Figure 3, Figure 4 and Figure 5.
