# Supplementary material for: One Is the Coldest Number: How Group Size and Body Weight Affect Thermal Preference in Weaned Pigs (3 to 15 kg)
Source: Animals (Basel). 2021 May 18;11(5):1447. doi: 10.3390/ani11051447 (PMC8157870; doi:10.3390/ani11051447)
Supplement: Supplementary file 1 [file animals-11-01447-s001.zip › animals-1182899-supplementary.pdf]

Supplementary Materials: The following are available online at www.mdpi.com/xxx/s1, Table S1: Ethogram for behaviors, Table S2: Ethogram for postures, Table S3 to S5: Statistical output, Figure S1: Image of conditioning box, Figure S2: Image of pig as sections used when coding video, and Figures S3 A-C demonstrate the equation use to create the regression models seen in Figures 3-5.

.

Table S1: Ethogram used for behavioral observations [13].

Category	Behavior	Description
Active	Active	Pig is walking about, can be seen actively engaged with the environment or with another pig. This includes fighting or head tossing with another piglet or interacting with troughs located on the long ends of the thermal apparatuses such as biting or chewing.
	Eating	Pigs head is in the feeding trough, located under heating elements (5 total), can only see back of head and ears while within in the feeding trough. All food troughs are located under the heating lamps.
	Drinking	Pigs head is in the watering trough, located opposite wall to heating elements (3 total), can only see back of head and ears while within in the watering trough. Water troughs are located on the opposite side of the thermal apparatus from the heating elements.
	Defecation	Pig is stationary or in a dog-sit position, can see fecal matter being excreted
Inactive		Pig is motionless and assumed to be sleeping. The animal may be inactive if sitting, standing or lying still and alert. An animal is stationary, slow and small head movements may be seen but their body is motionless.
Other		Pigs' behavior cannot be determined, camera angles or glare do not allow for accurate assessment.

Table S2: Ethogram used for posture observations [13].

Posture	Description
Upright	Pigs' body is erect and top line (back) is to the camera, includes pig standing on all four hoofs on ground and dog-sitting where pig has rump on the floor.
Sternal laying	Pig lies up-right with stomach and chest touching the ground, top line is facing the camera. This includes when a piglet is sternal on her anterior body and lateral on her posterior body. Sternal includes the medial plane of the head and body being perpendicular to a 45-degree angle to the ceiling.
Lateral laying	Pig lies on side with shoulder and rump touching the ground, top line is facing a wall. Medial plane of head and body are greater than 45 degrees and approximately 90 degrees to the ceiling.
Huddling	Pig is in contact with another piglet, with at least 50% of their bodies are touching. Pig can be sternal or lateral laying.
Other	Any other postures or those that cannot be determined, camera angles or glare do not allow for accurate assessment. When pig is in position transition and down on front knees but stays with hind end up for a while and may still be moving about.

Table S3. Statistical terms included in the cubic regression model which tested for differences in the percent of observations in various locations (temperatures) while pigs were inactive.

Parameters	F Value	*p*-Value
GroupID(Sex*GroupSize*WeightCat)	$F_{18,127} = 0.05$	1.000
Sex	$F_{1,127} = 0.00$	0.949
GroupSize	$F_{2,127} = 4.05$	*0.020
WeightCat	$F_{2,127} = 1.22$	0.298
Location	$F_{1,127} = 2.47$	0.119
Sex*GroupSize	$F_{2,127} = 0.01$	0.989
Sex*WeightCat	$F_{2,127} = 0.11$	0.900
GroupSize*WeightCat	$F_{4,127} = 0.09$	0.986
Location*GroupSize	$F_{2,127} = 6.90$	*0.001
Location*WeightCat	$F_{2,127} = 15.01$	*<0.001
GroupSize*WeightCat*Location	$F_{4,127} = 2.36$	0.057
Sex*GroupSize*WeightCat	$F_{4,127} = 0.24$	0.914
Location*Location	$F_{1,127} = 1.98$	0.162
Location*Location*GroupSize	$F_{2,127} = 4.00$	*0.021
Location*Location*WeightCat	$F_{2,127} = 1.48$	0.232
Location*Location*Location	$F_{1,127} = 0.85$	0.357
Location*Location*Location*WeightCat	$F_{2,127} = 5.23$	*0.007

* denote a significant difference ($p < 0.05$).

Table S4. Statistical terms included in the model which tested for differences in the percent of observations in various locations (temperatures) while pigs were huddling, groups tested as individuals has been excluded.

Parameters	F Value	*p*-Value
GroupID(Sex*GroupSize*WeightCat)	$F_{12,90} = 0.23$	0.996
Sex	$F_{1,90} = 2.08$	0.152
GroupSize	$F_{1,90} = 0.28$	0.599
WeightCat	$F_{2,90} = 2.08$	0.131
Location	$F_{1,90} = 7.09$	*0.009
Sex*GroupSize	$F_{1,90} = 0.00$	0.959
Sex*WeightCat	$F_{2,90} = 1.76$	0.178
GroupSize*WeightCat	$F_{2,90} = 0.28$	0.759
Location*GroupSize	$F_{2,90} = 0.58$	0.447
Location*WeightCat	$F_{2,90} = 2.71$	0.072
Sex*GroupSize*WeightCat	$F_{1,90} = 0.24$	0.7865
GroupSize*WeightCat*Location	$F_{1,90} = 0.19$	0.827

* denote a significant difference ($p < 0.05$).

Table S5. Statistical terms included in the model which tested for differences in the percent of observations in various locations (temperatures) while piglets were in various postures, huddling has been excluded.

**Parameters**	**F Value**	***p*-Value**
GroupID(Sex*GroupSize*WeightCat)	$F_{18,459} = 0.63$	0.875
Sex	$F_{1,459} = 5.43$	*0.020
GroupSize	$F_{2,459} = 10.40$	*<0.001
WeightCat	$F_{2,459} = 7.37$	*0.001
Posture	$F_{2,459} = 47.31$	*<0.001
Location	$F_{1,459} = 12.17$	*0.001
Sex*GroupSize	$F_{2,459} = 4.73$	*0.009
Sex*WeightCat	$F_{2,459} = 0.09$	0.916
GroupSize*WeightCat	$F_{4,459} = 1.14$	0.339
GroupSize*Posture	$F_{4,459} = 0.39$	0.816
GroupSize*Location	$F_{4,459} = 19.53$	*<0.001
WeigtCat*Posture	$F_{4,459} = 1.33$	0.257
WeightCat*Location	$F_{2,459} = 36.44$	*<0.001
Posture*Location	$F_{2,459} = 1.62$	0.200
Sex*GroupSize*WeightCat	$F_{2,459} = 1.89$	0.111
GroupSize*WeightCat*Location	$F_{4,459} = 8.30$	*<0.001
GroupSize*Posture*Location	$F_{4,459} = 1.61$	0.170
WeightCat*Posture*Location	$F_{4,459} = 11.52$	*<0.001
GroupSzie*WeightCat*Posture	$F_{8,459} = 1.24$	0.272
Location*Location	$F_{1,459} = 9.47$	*0.002
Location*Location*GroupSize	$F_{2,459} = 11.52$	*<0.001
Location*Location*WeightCat	$F_{2,459} = 4.50$	*0.012
Location*Location*Location	$F_{1,459} = 6.26$	*0.013
Location*Location*Location*WeightCat	$F_{2,459} = 9.81$	*<0.001

* denote a significant difference ($p < 0.05$).

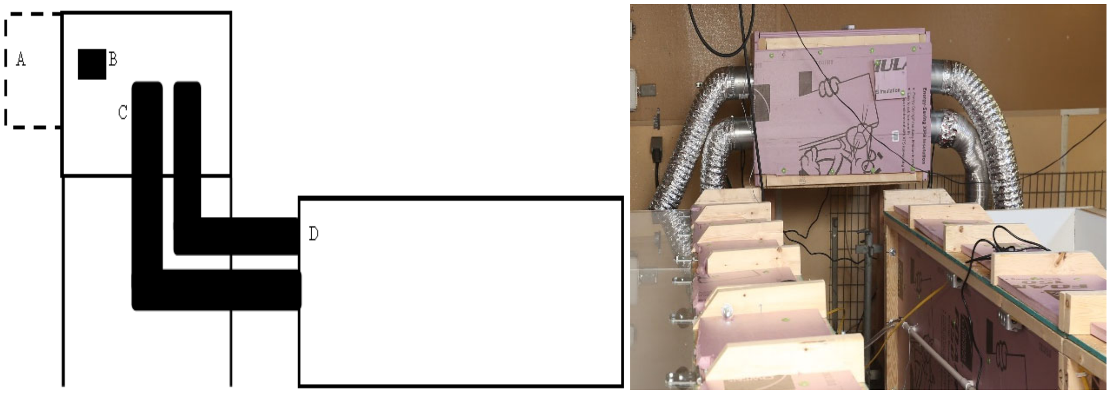

Figure S1: Image on the left displays the conditioning box used to push cold air into the thermal apparatuses, image on the right actual picture of the conditioning box. Here pictured is only one of the apparatuses. Legend: A) air conditioner, B) coolbot, C) ducts, D) thermal apparatus.

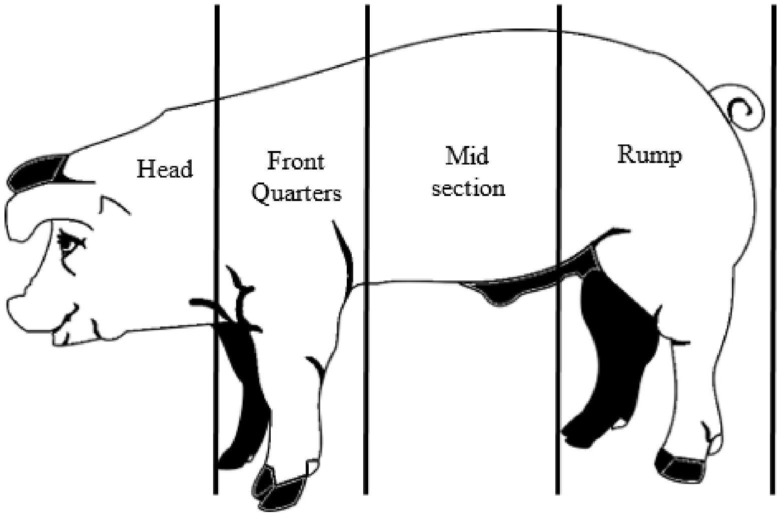

Figure S2: Diagram depicting sections of a pig used to assess percentage of body part located within a thermal section, each body section was equivalent to 25%. Head was considered from back of the ears to the snout, front quarters were considered back of the ears to behind the forelimbs, mid-section was from behind the forelimbs to front of the back limbs, and the rump was considered the front of the back limbs to base of the tail [13].

S 3A)

$$
\begin{aligned}
&0.7291889281\\
&+\text{Match}\left(\text{GroupSize}\right)\begin{pmatrix} 1 & \Rightarrow -0.107123199\\ 4 & \Rightarrow 0.0476670592\\ 2 & \Rightarrow 0.0594561396\\ \text{else} & \Rightarrow .\end{pmatrix}\\
&+ -0.01926068 \bullet \text{Temperature}\\
&+\text{Match}\left(\text{GroupSize}\right)\begin{pmatrix} 1 & \Rightarrow \left(\text{Temperature} - 24.418\right)\bullet 0.0215604468\\ 4 & \Rightarrow \left(\text{Temperature} - 24.418\right)\bullet -0.009830977\\ 2 & \Rightarrow \left(\text{Temperature} - 24.418\right)\bullet -0.01172947\\ \text{else} & \Rightarrow .\end{pmatrix}\\
&+\left(\text{Temperature} - 24.418\right)\bullet\left(\left(\text{Temperature} - 24.418\right)\bullet 0.0015778529\right)\\
&\left(\text{Temperature} - 24.418\right)\\
&+\bullet\left(\left(\text{Temperature} - 24.418\right)\bullet \text{Match}\left(\text{GroupSize}\right)\begin{pmatrix} 1 & \Rightarrow 0.0044011141\\ 4 & \Rightarrow -0.00161496\\ 2 & \Rightarrow -0.002786154\\ \text{else} & \Rightarrow .\end{pmatrix}\right)
\end{aligned}
$$

S 3B)

$$0.7291889281$$

$$+\text{Match}(\text{WgtCat})\begin{pmatrix}\text{"L"} \Rightarrow 0.0408059145 \\ \text{"M"} \Rightarrow 0.0165020453 \\ \text{"S"} \Rightarrow -0.05730796 \\ \text{else} \Rightarrow .\end{pmatrix}$$

$$+ -0.01926068 \bullet \text{Temperature}$$

$$+\text{Match}(\text{WgtCat})\begin{pmatrix}\text{"L"} \Rightarrow (\text{Temperature} - 24.418)\bullet -0.094787863 \\ \text{"M"} \Rightarrow (\text{Temperature} - 24.418)\bullet 0.0531640776 \\ \text{"S"} \Rightarrow (\text{Temperature} - 24.418)\bullet 0.041623785 \\ \text{else} \Rightarrow .\end{pmatrix}$$

$$+(\text{Temperature} - 24.418)\bullet((\text{Temperature} - 24.418)\bullet 0.0015778529)$$

$$+\begin{matrix}(\text{Temperature} - 24.418) \\ \bullet\left((\text{Temperature} - 24.418)\bullet \text{Match}(\text{WgtCat})\begin{pmatrix}\text{"L"} \Rightarrow -0.001333101 \\ \text{"M"} \Rightarrow -0.001395455 \\ \text{"S"} \Rightarrow 0.0027285555 \\ \text{else} \Rightarrow .\end{pmatrix}\right)\end{matrix}$$

$$+\begin{matrix}(\text{Temperature} - 24.418) \\ \bullet\left((\text{Temperature} - 24.418)\bullet((\text{Temperature} - 24.418)\bullet 0.0003378197)\right)\end{matrix}$$

$$+\begin{matrix}(\text{Temperature} - 24.418) \\ \bullet\left(\begin{matrix}(\text{Temperature} - 24.418) \\ \bullet\left((\text{Temperature} - 24.418)\bullet \text{Match}(\text{WgtCat})\begin{pmatrix}\text{"L"} \Rightarrow 0.0016461048 \\ \text{"M"} \Rightarrow -0.001075324 \\ \text{"S"} \Rightarrow -0.000570781 \\ \text{else} \Rightarrow .\end{pmatrix}\right)\end{matrix}\right)\end{matrix}$$

S 3C)

```
-0.427164753
+ Match( WgtCat )
    "L"    ⇒ 0.1986244941
    "M"    ⇒ -0.041064739
    "S"    ⇒ -0.157559755
    else   ⇒ .
+ Match( Posture )
    "Lateral"  ⇒ -0.211602121
    "Sternal"  ⇒ -0.131305575
    "Upright"  ⇒ 0.342907696
    else       ⇒ .
+ -0.06192594 • Temperature
+ Match( WgtCat )
    "L" ⇒ Match( Posture )
        "Lateral"  ⇒ 0.0929767437
        "Sternal"  ⇒ 0.0113496361
        "Upright"  ⇒ -0.10432638
        else       ⇒ .
    "M" ⇒ Match( Posture )
        "Lateral"  ⇒ -0.030328559
        "Sternal"  ⇒ -0.009381833
        "Upright"  ⇒ 0.039710392
        else       ⇒ .
    "S" ⇒ Match( Posture )
        "Lateral"  ⇒ -0.062648185
        "Sternal"  ⇒ -0.001967803
        "Upright"  ⇒ 0.0646159878
        else       ⇒ .
    else ⇒ .
+ Match( WgtCat )
    "L"  ⇒ ( Temperature - 24.418 ) • -0.213441529
    "M"  ⇒ ( Temperature - 24.418 ) • 0.1237421692
    "S"  ⇒ ( Temperature - 24.418 ) • 0.0896993599
    else ⇒ .
+ Match( Posture )
    "Lateral"  ⇒ ( Temperature - 24.418 ) • 0.014830533
    "Sternal"  ⇒ ( Temperature - 24.418 ) • -0.00946216
    "Upright"  ⇒ ( Temperature - 24.418 ) • -0.005368373
    else       ⇒ .
+ Match( WgtCat )
    "L" ⇒ Match( Posture )
        "Lateral"  ⇒ ( Temperature - 24.418 ) • -0.043846298
        "Sternal"  ⇒ ( Temperature - 24.418 ) • -0.034605133
        "Upright"  ⇒ ( Temperature - 24.418 ) • 0.0784514315
        else       ⇒ .
    "M" ⇒ Match( Posture )
        "Lateral"  ⇒ ( Temperature - 24.418 ) • 0.034797393
        "Sternal"  ⇒ ( Temperature - 24.418 ) • 0.008119947
        "Upright"  ⇒ ( Temperature - 24.418 ) • -0.04291734
        else       ⇒ .
    "S" ⇒ Match( Posture )
        "Lateral"  ⇒ ( Temperature - 24.418 ) • 0.0090489052
        "Sternal"  ⇒ ( Temperature - 24.418 ) • 0.0264851863
        "Upright"  ⇒ ( Temperature - 24.418 ) • -0.035534092
        else       ⇒ .
    else ⇒ .
+ ( Temperature - 24.418 ) • ( Temperature - 24.418 ) • 0.0050026634
+ ( Temperature - 24.418 ) • ( Temperature - 24.418 ) • Match( WgtCat )
    "L"  ⇒ -0.006037022
    "M"  ⇒ 0.0001398299
    "S"  ⇒ 0.0058971926
    else ⇒ .
+ ( Temperature - 24.418 ) • ( Temperature - 24.418 ) • ( Temperature - 24.418 ) • 0.0013242567
+ ( Temperature - 24.418 ) • ( Temperature - 24.418 ) • ( Temperature - 24.418 ) • Match( WgtCat )
    "L"  ⇒ 0.0032515852
    "M"  ⇒ -0.002184375
    "S"  ⇒ -0.00106721
    else ⇒ [illegible]
```

Figure S3: The following equations were taken from our statistical model to create the predicted curves for A) Figure 3 observations of inactive behavior per group size; B) Figure 4 observations of inactive per weight category; C) Figure 5A-5C for posture observations (lateral, sternal, and upright) per weight category.
